# Stability of Yellow Fever Virus under Recombinatory Pressure as Compared with Chikungunya Virus

**DOI:** 10.1371/journal.pone.0023247

**Published:** 2011-08-03

**Authors:** Charles E. McGee, Konstantin A. Tsetsarkin, Bruno Guy, Jean Lang, Kenneth Plante, Dana L. Vanlandingham, Stephen Higgs

**Affiliations:** 1 Department of Pathology, University of Texas Medical Branch, Galveston, Texas, United States of America; 2 Carolina Vaccine Institute, The University of North Carolina, Chapel Hill, North Carolina, United States of America; 3 Sanofi Pasteur, Campus Mérieux, Marcy-L'étoile, France; University of Texas Medical Branch, United States of America

## Abstract

Recombination is a mechanism whereby positive sense single stranded RNA viruses exchange segments of genetic information. Recent phylogenetic analyses of naturally occurring recombinant flaviviruses have raised concerns regarding the potential for the emergence of virulent recombinants either post-vaccination or following co-infection with two distinct wild-type viruses. To characterize the conditions and sequences that favor RNA arthropod-borne virus recombination we constructed yellow fever virus (YFV) 17D recombinant crosses containing complementary deletions in the envelope protein coding sequence. These constructs were designed to strongly favor recombination, and the detection conditions were optimized to achieve high sensitivity recovery of putative recombinants. Full length recombinant YFV 17D virus was never detected under any of the experimental conditions examined, despite achieving estimated YFV replicon co-infection levels of ∼2.4×10^6^ in BHK-21 (vertebrate) cells and ∼1.05×10^5^ in C_7_10 (arthropod) cells. Additionally YFV 17D superinfection resistance was observed in vertebrate and arthropod cells harboring a primary infection with wild-type YFV Asibi strain. Furthermore recombination potential was also evaluated using similarly designed chikungunya virus (CHIKV) replicons towards validation of this strategy for recombination detection. Non-homologus recombination was observed for CHIKV within the structural gene coding sequence resulting in an in-frame duplication of capsid and E3 gene. Based on these data, it is concluded that even in the unlikely event of a high level acute co-infection of two distinct YFV genomes in an arthropod or vertebrate host, the generation of viable flavivirus recombinants is extremely unlikely.

## Introduction

The family Flaviviridae contains three genera of viruses of significant human and veterinary importance: *Hepacivirus* (Human Hepatitis C virus), *Pestivirus* (bovine viral diarrhea virus (BVDV) and classical swine fever virus), and *Flavivirus*. The flaviviruses, genus *Flavivirus* can be further subdivided into those viruses which are transmitted by tick (tick-borne flaviviruses), mosquitoes (mosquito-borne flaviviruses), and those with no known vector [1]. The mosquito-borne flaviviruses, for example yellow fever virus (YFV), and the four dengue virus serotypes (DENV 1–4), are responsible for millions of infections and thousands of deaths annually [2]–[5].

The flavivirus genome is ∼11–12 Kb, which is comprised of a positive sense single stranded RNA molecule that contains a 5′methylguanosine cap but no poly-adenosine tail. This genome contains one open reading frame (ORF), encoding three structural proteins; (capsid (C), premembrane (prM), and envelope (E)) and seven nonstructural proteins (NS1, NS2A, NS2B, NS3, NS4A, NS4B, and NS5), flanked by 5′ and 3′ untranslated regions (UTR). The flavivirus virion is ∼50 nm in diameter and is comprised of an electron dense nucleocapsid core surrounded by an endoplasmic reticulum derived lipid bilayer [6]–[8]. Within this lipid bilayer are embedded the mature membrane and E proteins. The E protein has three distinct domains; a receptor binding domain, a central linker domain, and a dimerization domain which contains the membrane fusion loop [9]–[11]. The flavivirus virion contains 180 E proteins associated as dimers that are oriented parallel to the lipid bilayer in an icosaheldral symmetry [12].

Certain viruses belonging to the family Flaviviridae, principally non-arthropod-borne members, have long been known to be competent to undergo recombination. The seminal observation of Flaviviridae recombination involved detection, by Northern blot analysis, of cellular ubiquitin sequences which had been incorporated into the BVDV genome [13]. The subsequent development of pathogenic mucosal disease was always characterized by the simultaneous presence of two distinct BVDV biotypes classified as cytopathic (cp) and non-cytopathic (ncp) based on cell culture phenotype [14]. Sequence analysis of viral pairs, isolated from fatal bovine infections, identified recombination-driven sequence duplications, deletions, insertions and rearrangements within ncp-variants. However, >99% sequence identity was detected between matched cp and ncp variants when the non-recombinant regions were compared [15]–[18]. Furthermore, molecular characterizations of isolates derived from animals exhibiting post-vaccination mucosal disease suggest that recombination can occur between ncp-BVDV wt and cp-BVDV vaccines in persistently infected animals [19], [20].

Hepatitis C virus (HCV; genus *Hepacivirus*) is capable of initiating a persistent hepatatropic infection in humans. The seminal observation of HCV recombination involved the detection of a 2k/1b recombinant (RF1_2k/1b) in human serum samples collected in St. Petersburg, Russia [21]. This recombinant has been repeatedly isolated from human serum samples across a wide geographic range, including: Russia, Ireland, Estonia, and Uzbekistan [22]–[25]. Sequence analysis of these related isolates suggests that they are all derived from a common ancestor which likely recombined 50–80 years ago and subsequently spread over a large geographic range [25]. Furthermore, three other distinct inter-typic recombinant viruses have been identified: a 2i/6p Vietnamese isolate [26], a 2b/1b Philippine isolate [27]; a 2(unknown subtype)/5a French isolate [23] and there are reports of intra-typic recombination [28]–[30].

Until recently, evidence of recombination within the members of *Flavivirus* genus was based solely on phylogenetic sequence analyses. Such studies suggested that recombinant intra-typic DENV exist across all four serotypes [31]–[37]. Cross-over regions appear to be localized to the 5′ end of the genome (prM, E, and NS1) [31]–[36]. One report identified recombination in the DENV 1 NS3 gene sequence [37]. Perhaps the most interesting aspect of these analyses has been the identification of DENV genomes that appear to be mosaics resulting from multiple homologous recombinant events [31], [33], [35], [36]. One report suggested that a sequenced isolate of DENV 1 contains three recombinant regions coincident with six homologous polymerase jumps [37]. Intra-typic recombination has been reported for Japanese encephalitis virus (JEV) and St. Louis encephalitis virus (SLEV) [36] with cross-over sequences localized to the E gene coding sequence. Additionally, a recent report by Pickett and Lefkowitz (2009) has also identified a recombinant West Nile virus (WNV) sequence with the cross-over occurring in the NS5 polymerase gene [38]. However it should be noted, that in none of these studies was recombinant clonally isolated or characterized. Furtheremore, naturally occurring recombinant mosquito-borne/tick-borne flaviviruses have never been recovered for characterization under laboratory conditions. With the exception of a recent report by Taucher et al., (2009) [39], all previous reports of flavivirus recombination have involved analysis of sub-cloned PCR generated amplicons and/or sequences deposited in databases. As such these reports have met with skepticism due to a lack of empirical to substantiate recombinant events and secondary analyses which have suggested that some “recombinant” sequences may be artifacts of incorrectly annotated or assembled sequence files [40]–[42]. Furthermore, if recombination has occurred for some of these viruses such as SLEV no evidence exists to sugest this has had any significant impact on the viral epidemiology or ecology.

The changing global pattern of human activity, including increased travel and encroachment into sylvatic habitat, coupled with recent explosions in competent vector populations have dramatically increased the global distributions of many of these viruses. WNV and SLEV are now both widely distributed across North America and share a transmission cycle involving *Culex* mosquitoes and passerine birds. Furthermore, the rapid global expansion of the DENV 1–4 [3] has lead to *Aedes* mosquito driven co-circulation of all four serotypes coincident with YFV transmission in large areas of South America and Africa. As a consequence, a number of flaviviruses with similar ecological niches now have sympatric geographic regions of endemic/epidemic transmission, dramatically increasing the potential for arthropod vectors and/or vertebrate hosts to become co-infected with multiple viruses. The use of live attenuated flavivirus vaccines, such as the YFV 17D vaccine and 17D derived chimeric vaccines for example the ChimeriVax™ platform [43], to control disease in regions of epidemic/endemic transmission has been postulated to represent a risk for co-infection and potential recombination [33], [34], [44]. Since the propensity of factors that might facilitate flavivirus-flavivirus co-infection interactions continues to rise, investigations into the potential for the generation of wild-type (wt)/wt and wt/vaccine recombinants is important for our understanding, and ability to, predict how these viruses are likely to evolve if introduced into natural transmission cycles. Experiments described in this paper were designed to evaluate the potential of YFV 17D to undergo homologous or non-homologous recombination. However, as flavivirus recombination potential, which appears to be very inefficient and potentially virus specific, [39] has only just begun to be elucidated under laboratory conditions we evaluated intra-genic recombination using similarly designed CHIKV replicons. Given our established expertise with CHIKV reverse genetics systems [45]–[47], the known propensity for alphaviruses to recombine [48]–[52], and the similar natural transmission cycle (*Aedes* mosquito to human) CHIKV appeared to be an ideal system to serve as a positive recombination control to comparatively analyze and optimize the relative efficiencies of intra-genic RNA arthropod-borne virus recombination.

## Materials and Methods

### Cells

Baby hamster kidney (BHK) cells were maintained in alpha-minimal essential medium (αMEM; Invitrogen, Carlsbad, CA) supplemented with 10% fetal bovine serum (FBS; Invitrogen, Carlsbad, CA), 100 U/mL penicillin 100 µg/mL streptomycin (pen-strep), L-glutamine (L-glu; Cellgro® Mediatech, Inc.) and vitamins and maintained at 37°C with 5% CO_2_. African green monkey kidney (Vero) cells were grown in minimum essential medium (MEM; Invitrogen, Carlsbad, CA) with 2% bovine growth serum (Hyclone, Logan, UT), pen-strep, non-essential amino acids (Sigma-Aldrich, St. Louis, MO), and L-glu and maintained at 37°C in the presence of 5% CO_2_. *Aedes albopictus* (C6/36) cells were grown in Liebovitz L-15 media with 10% FBS, 10% tryptose phosphate broth (Sigma-Aldrich, St. Louis, MO), pen-strep, and 1% L-glu and maintained at 28°C. *Aedes albopictus* (C_7_10) cells were grown in Dulbecco's minimum essential medium (Gibco, Carlsbad, CA) with 10% FBS, pen-strep, L-glu, and maintained at 30°C in the presence of 5% CO_2_. Vero cells used for electroporation were grown in MEM with 5% FBS and pen-strep and maintained at 37°C.

### Plasmid Constructs

#### Alphavirus plasmid constructs

**Figure 1:**
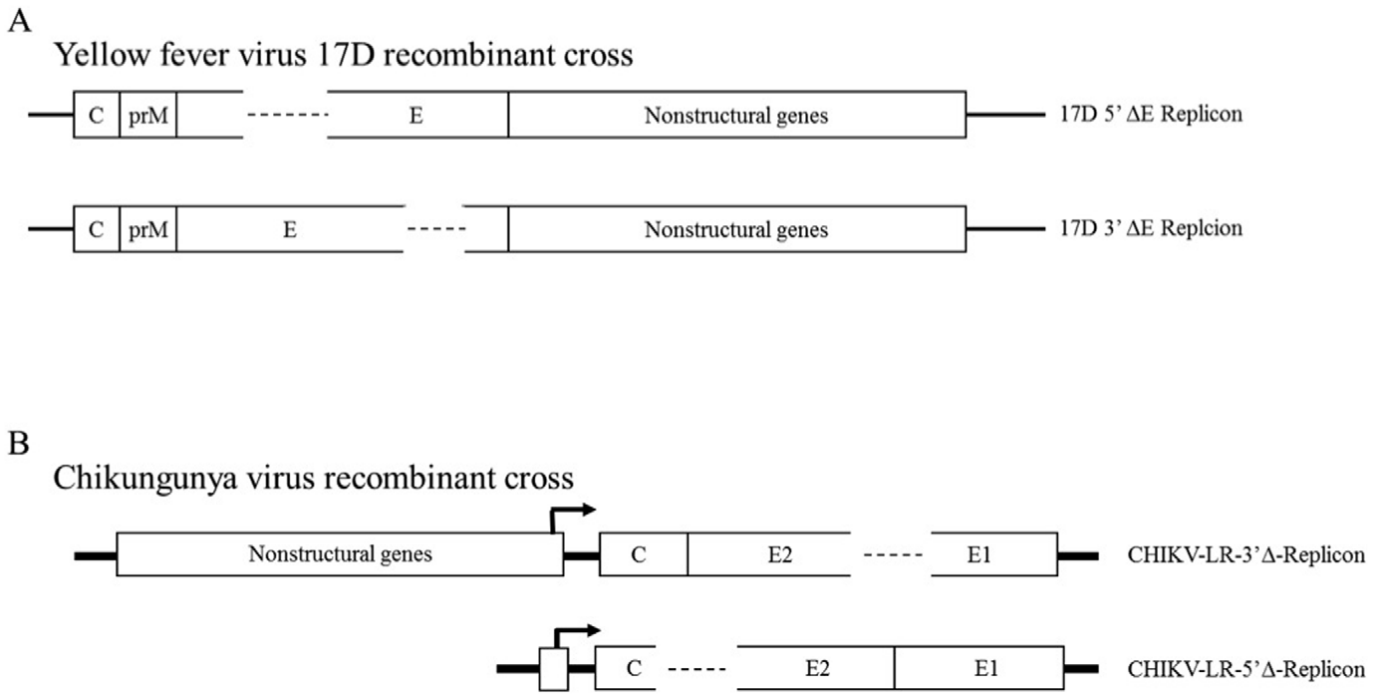
Schematic representation of flavivirus and alphavirus recombinant crosses. A) Yellow fever virus 17D deletion mutant replicon recombinant cross, B) chikungunya virus deletion mutant replicon/defective helper recombinant cross.

All plasmid constructs or infectious clones (IC) containing replicon, defective helper, and full length viral genomes were generated using standard polymerase chain reaction (PCR) based cloning methodologies [53]. To evaluate intra-genic homotypic recombination of CHIKV, a replicon/defective helper system was designed which contained complementary frame shift deletions (ΔFS), separated by 967 nt of homologus sequence, within the structural coding cassette. ΔFS engineered into the CHIKV structural ORF were chosen based on ease of cloning. A 3′ ΔFS of nt 9079–9191 in the E2 sequence was engineered into both the CHIKV-LR IC [45] and a CHIKV-LR-5′ Cherry fluorescent protein expressing IC (Higgs lab, unpublished) to generate replicon genomes CHIKV-LR-3′Δ-Rep and CHIKV-LR-3′Δ-Cherry-Rep respectively (**Figure 1B**). Additionally, a complementary 5′ ΔFS of nt 7910–8092 in the capsid sequence was engineered into both the CHIKV-LR IC and CHIKV-LR-5′ GFP IC [45] in concert with a major deletion of 6,897 nt of the CHIKV nonstructural cassette to generate defective helper genomes CHIKV-LR-5′Δ-Help and CHIKV-LR-5′Δ-GFP-Help respectively. Plasmid maps, primer sequences, and detailed cloning methodologies available from the authors upon request.

#### Flavivirus plasmid constructs

All YFV 17D plasmid constructs were generated, or derived from, the parental YFV 17D IC (pACNR/FLYF-17D; [54]). This construct was further modified in a two step cloning process to generate p17D RBZ IC which contained a silent mutation at nucleotide (nt) 495 to allow for incorporation of a unique BspE*I* and a Hepatitis Δ rybozyme sequence immediately downstream of the viral sequence. Two distinct 17D deletion mutant virus variants designated 17D 5′ ΔE and 17D 3′ ΔE respectively, were constructed to permit evaluation of YFV intra-genic heterotypic recombination (**Figure 1A**). Each of these mutants contained an in-frame deletion in the E protein coding sequence. Briefly, a 5′ deletion of nt 1109–1324 was engineered into p17D RBZ IC to generate p17D-5′-ΔE-Rep and a complementary 3′ deletion of nt 1984–2236 was also engineered into p17D RBZ IC to generate p17D-3′-ΔE-Rep resulting in the generation of two complementary deletions separated by 636 nt of homologous coding sequence. Additionally, the parental YFV Asibi IC (pYFV-As IC) as described by [55] was also modified by incorporation of this RBZ sequence to generate pYFV-As RBZ IC.

A 5′ fluorescent protein reporter cassette was also fused within the viral open reading frame of each of the aforementioned YFV IC genomes, comprised of 25 amino acids (aa) of the YFV capsid sequence followed by the sequences for the reporter and a foot-and-mouth disease virus 2A protease immediately fused to a human codon optimized full length YFV capsid, sequence as previously described [56]. Insertion of reporter cassettes into flavivirus genomes in this way facilitated efficient exogenous gene expression *in vitro* and *in vivo* with increased stability as compared to classic 3′ insertions [56], [57]. Reporter cassettes expressing either enhanced green fluorescent protein (GFP) or Cherry fluorescent protein (Cherry) were engineered into the YFV constructs including p17D RBZ IC, pYFV-As RBZ IC, and p17D 5′/3′ ΔE replicons to generate plasmid constructs p17D-GFP-RBZ IC, p17D-Cherry-RBZ IC, pYFV-As-GFP-RBZ, p17D-3′-ΔE-GFP-Rep and p17D-5′-ΔE-Cherry-Rep. Additionally, a YFV 17D packaging construct was constructed, to facilitate *trans* packaging of envelope protein sequence deletion mutants, by replacing the GFP coding sequence of a VEEV-GFP replicon, previously described by [58], with the sequences for the YFV 17D structural genes.

### Generation, isolation, and characterization of clonal recombinant genomes

The basic experimental design for the generation and cell culture purification of full length recombinant genomes was as follows. Viral RNAs were *in vitro* transcribed from linearized plasmid genomes using the mMESSAGE mMACHINE Sp6 Kit (Ambion, Applied Biosystems, Foster City, CA) followed by LiCl precipitation in accordance with manufacturer protocols. Reaction yield was then quantified using a SmartSpec Plus spectrophotometer and known quantities of RNA were electroporated, either individually (negative controls) or as mixtures of specific ratios (recombinant crosses as ten-fold serial dilutions), into either BHK-21 or C_7_10 cells. For all recombination experiments, immediately after electroporation, cells were resuspended in 5.0 mL of appropriate maintenance media and 1/5 of the total electroporation suspension (∼2×10^6^ cells), with the addition of 1.0 mL of sterile maintenance medium, plated in a single well of a six-well plate and incubated at 37°C or 30°C for 24 h (alphavirus experiments) to 72 h (flavivirus experiments). At the time of electroporation, infectious centers assays [45] were performed for those constructs engineered to contain fluorescent protein expression cassettes, to permit visual estimation of co-infection levels within each experimental set. At 24 h or 72 h post-electroporation, cell culture supernatant was removed and stored at −80°C. Additionally, total cellular RNA was harvested using TRIzol® Reagent (Invitrogen, Carlsbad, CA) according to the manufacturer's protocol.

Clonal populations of recombinant genomes were isolated via infection of 1×10^7^ Vero cells in suspension with 500 µL of electroporation supernatant samples, with agitation for 1 h at room temperature. Following this incubation, cells were seeded into 96-well plates (∼3.5×10^4^ cells per well; 150 µL total volume per well) and incubated for 72 h at 37°C at which time all wells were examined visually. For CHIKV intra-genic recombinant crosses, total medium from those wells identified as positive for cytopathic effects (CPE) was harvested and stored at −80°C for additional analysis. Due to the non-cytopathic nature of YFV 17D, especially when inoculated at a low multiplicity of infection (moi), at 72 h post-infection all medium was removed from primary isolation plates and placed in correspondingly labeled 96-well plates and stored at −80°C. Plates were then fixed with acetone∶PBS (3∶1) and stained using an indirect immunofluorescence assay. Briefly, plates were rehydrated with 1× PBS followed by sequential 40 m incubations at 37°C with 1∶500 concentration of a YFV NS1 specific murine monoclonal antibody designated 86.3a [59] and a 1∶500 concentration of a goat-anti mouse IgG human absorbed secondary antibody (Alexa Fluor® 488 Dye; Invitrogen, Carlsbad, CA). All wells were visually examined for the presence of YFV 17D infectious foci using an Olympus IX-50 epifluorescence microscope.

All clonal recombinant isolates were amplified by a single passage in C6/36 cells and, following a 48 h incubation, supernatants were harvested for each designated recombinant stock and stored at −80°C until analyzed for the presence of infectious virus by plaque assay. Additionally, RNA was extracted from supernatant samples QIAamp® Viral RNA Mini Kit (Qiagen, Valencia, CA) and C6/36 (TRIzol Reagent) cells and subjected to sequence and Northern blot analysis respectively.

#### Virus quantification

Infectious virus samples were titered by plaque assay as follows. Briefly, Vero cells were seeded in well plates at ∼70% confluence and allowed to attach and grow for 4–6 h, directly inoculated with ten-fold serial dilutions of viral samples and following a 1 h incubation overlayed with an agarose overlay (alphavirus samples) or semi-solid carboxy methyl-cellulose overlay (flavivirus samples). Alphavirus plaque assay plates were then incubated at 37°C for 48–72 h (depending on the specific virus being assayed), fixed with 3.8% formaldehyde solution, stained with crystal violet, and scored, whilst flavivirus samples were fixed and stained as above. Titers were calculated as plaque forming units (pfu)/mL. Infectious virus samples assayed by virus titration were as previously described [60]. Briefly, ten-fold serial dilutions of virus samples were performed in a 96-well plate format and seeded with Vero cells. Titers were calculated as log_10_ tissue culture infectious dose 50 (log_10_ TCID_50_) per mL.

#### Northern blot

The presence of full length recombinant viral genomes cellular RNA was assessed by Northern blot using the NorthernMax®-Gly glyoxal-based system for Northern blots (Ambion, Applied Biosystems, Foster City, CA) and BrightStar®-Plus membrane (Ambion) in accordance with manufacturer protocols. RNA bound membranes were then probed with a virus specific biotinylated 40 nt oligonucleotide probe (CHIKV-5′UTR BioProbe; 5′-CACGTACACAGGATCCATGATGGGTTATTAATCTC TTGCT) at a concentration of 1.5 pM overnight at 42°C. Mixtures of virus specific synthetic RNAs were generated by mixing equimolar amounts of *in vitro* transcribed purified viral RNAs derived from plasmids of varying lengths; i.e. full length ∼12.0 Kb, replicon ∼8.0 Kb, defective helpers ∼2.0–5.0 Kb and included as molecular size markers. All Northern blot membranes were developed using the BrightStar® BioDetect™ nonisotopic detection kit (Ambion) in accordance with manufacturers protocols and chemiluminescence was detected by exposing the treated membrane to Kodak Biomax XAR film (Sigma-Aldrich, St. Louis, MO).

#### Sequencing of putative recombinants

Two clonal isolates, per recombination experiment, from the lowest dilution in which recombination was detected (minimum level of co-infection) were selected for sequence analysis of the putative junction/cross-over region. Briefly, RNA was isolated from the recombinant stocks using the QIAamp viral RNA extraction and cDNA generated using the SuperScript III First-Strand Synthesis kit (Invitrogen, Carlsbad, CA). cDNA was then PCR amplified and amplicons cloned into pBluescript SK+ (Stratagene, La Jolla, CA). The EcoR*V* site was chosen to facilitate efficient cloning of blunt end fragments between the T_7_ and T_3_ promoter/primer binding sites to facilitate ease of insert detection and sequencing. Sequencing reactions were performed at the University of Texas Medical Branch (UTMB) Protein Chemistry Core and sequence files were edited and analyzed using the GeneRunner 3.05 software suite.

## Results

### Analysis of CHIKV intra-genic recombination

To validate that our strategy of using viral genomes containing intra-ORF deletions was indeed capable of detecting intra-genic recombination of positive sense single stranded RNA viruses, the relative efficiency of CHIKV intra-genic recombination was evaluated using a replicon/defective helper system that contains complementary 5′ and 3′ deletions in the structural ORF (Fig. 1B). RNA from pCHIK-LR-3′Δ-Rep, containing a 112 nt FSΔ in E2, was co-electroporated against ten-fold serial dilutions of RNA from pCHIK-LR-5′Δ-Help, which possesses a 182 nt FSΔ in capsid. RNAs derived from pCHIK-LR-3′Δ-Cherry-Rep and pCHIK-LR-5′Δ-GFP-Help were also co-electroporated in parallel (10 µg per RNA species) to verify co-infection efficiency within this experimental set, 1.2×10^7^/10 µg each RNA.

**Figure 2:**
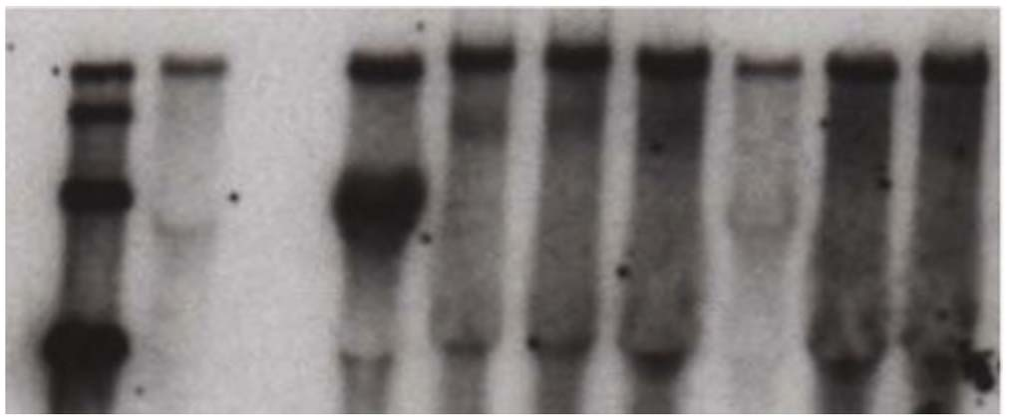
Northern blot analysis of chikungunya virus (CHIKV) intra-genic recombinant isolates. Blot was hybridized with CHIKV-5′UTR BioProbe. Lane 1: CHIKV RNA ladder (size markers indicate CHIKV RNAs of full length genomic, replicon, full length structural gene helper, and capsid only structural helper respectively), Lane 2: CHIKV-LR-3′Δ-Replicon only electroporation, Lane 3: CHIKV-LR-5′Δ-Helper only electroporation, Lanes 4 and 8: CHIKV-LR-3′Δ-Replicon/ CHIKV-LR-5′Δ-Helper co-electroporations, and Lanes 5, 6, 7, 9, and 10: clonal recombinant isolates.

Recombination was observed to occur within the 967 nt of the structural ORF under investigation to a minimum of ∼10^5^ co-infections. Clonal isolates of intra-genic recombinant CHIKV could be directly purified from co-electroporation dilutions 10^6^ (n = 3 isolates) and 10^5^ (n = 2 isolates). CHIKV intra-genic recombinant isolates were competent for replication in both Vero and C6/36 cells and reached average titers of 2.96×10^7^ pfu/mL by 48 h post-infection in C6/36 cells, which are comparable to titers observed post-infection with infectious clone derived wt CHIKV. It is important to note that CHIK-LR-5′Δ-Rep is only 112 nt smaller in size than the CHIK-LR wt virus, and so size shift analysis as applied in Sindbis virus recombination experiments [50], [51] was not possible in our experiments to verify the presence of virus specific intra-genic recombinant RNAs. However, Northern blot analysis (CHIKV-5′UTR BioProbe) of all CHIKV intra-genic recombinants successfully identified virus specific RNAs of full-length-like size in C6/36 total cellular RNA samples 48 h post infection with putative intra-genic recombinants (**Figure 2** **; Lanes 5, 6, 7, 9, and 10**).

**Figure 3:**
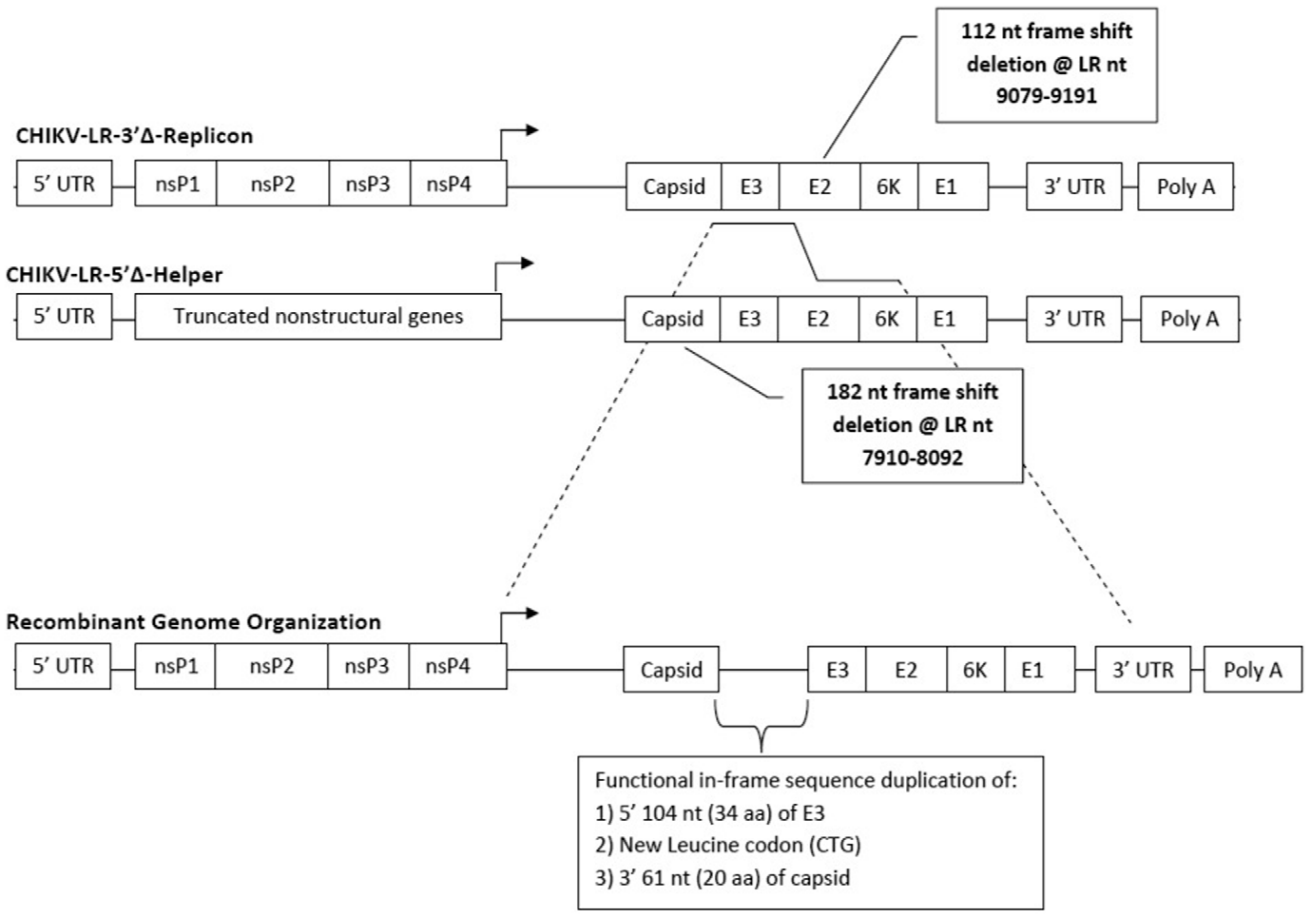
Schematic representation of parental replicon/defective helper recombinant cross (CHIK-LR-3′Δ-Replicon and CHIK-LR-5Δ′-Helper) along with sequence topology analysis of the cross-over region and resulting recombinant topology of clonally isolated recombinant genomes. Genes not necessarily shown to scale. aa-amino acid, E-envelope glycoprotein, ns-nonstructural, nt-nucleotide, LR-LaReunion, and UTR-untranslated region, @LR location of sequence deletion.

The putative cross-over sequence, and entire region between the engineered 5′/3′ ΔFS, of two isolates from co-infection dilution ∼10^5^ was sequenced to determine the resulting gene topology. Recombination appeared to have occurred between the two ΔFS mutations as expected, however, the nature of this recombination was non-homologous and resulted in the generation of a stable in-frame duplication of structural gene coding sequence (**Figure 3**). This duplication was comprised of the 5′ 34 amino acids (aa) (104 nt) of the CHIK-LR-5′Δ-Rep E3 coding sequence followed by the 3′ 20aa (61 nt) of the CHIK-LR-3′Δ-Help capsid sequence and the generation of a new hybrid leucine codon (CTG) at the jump point. This topology likely resulted in the retention of the capsid-E3 cleavage signal sequences allowing for efficient removal of the duplicated partial proteins during structural gene transcription and translation, thus allowing for the efficient expression of full length functional C and E3. This topology remained stable over five blind passages in Vero cells with no evidence of sequence evolution or loss of duplicated structural gene sequences.

### *In vitro* analysis of YFV 17D and Asibi full length and replicon growth kinetics

**Figure 4:**
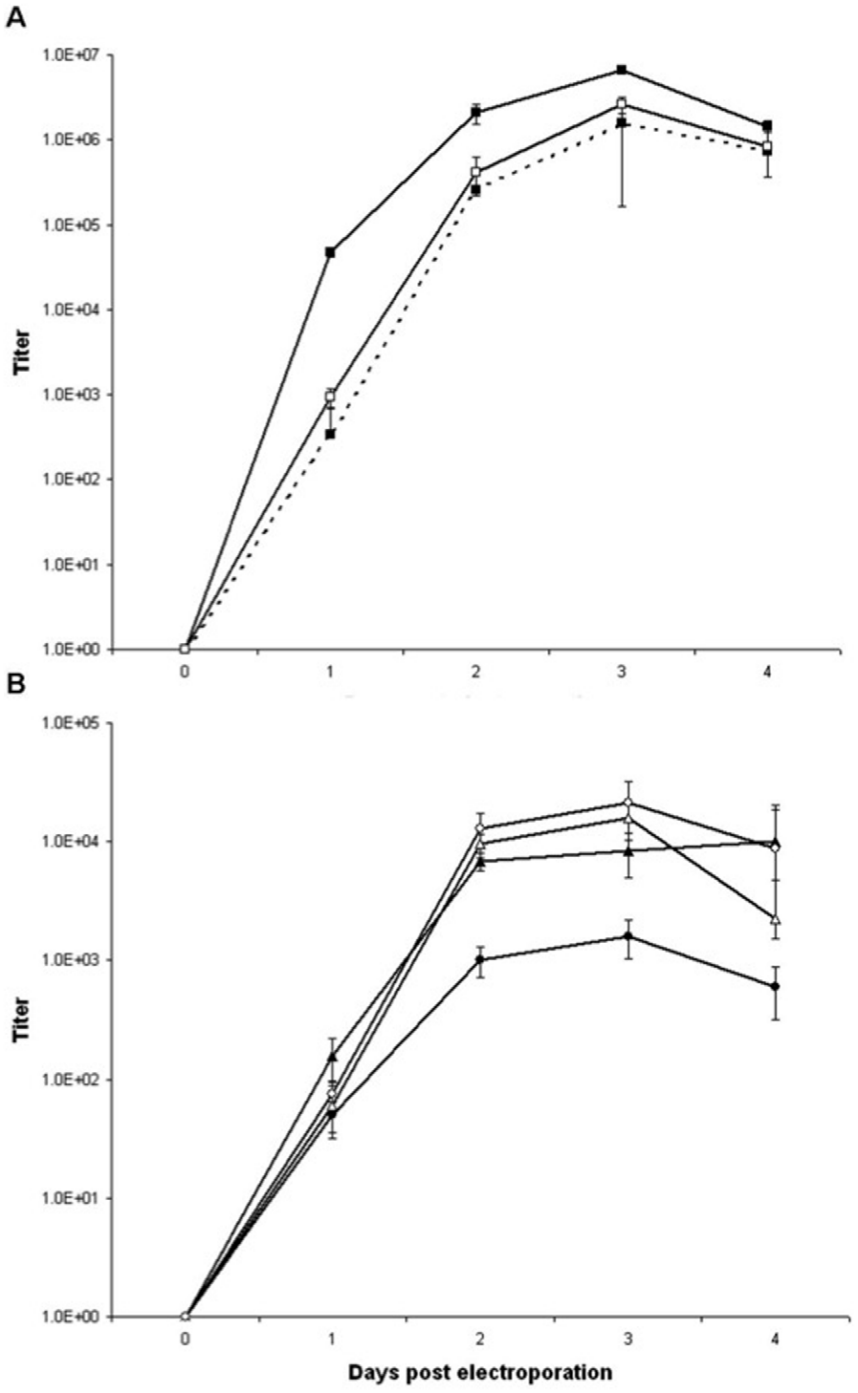
Comparison of post-electroporation growth kinetics of yellow fever viruses (YFV) and deletion mutants in BHK-21 cells. A) YFV 17D (▪ solid line), YFV 17D GFP (▪ dashed line), and YFV 17D Cherry (○ solid line); B) YFV 17D 5′ ΔE (▴), YFV 17D 3′ ΔE (•), YFV 17D 5′ΔE Cherry (Δ), and YFV 17D 3′ ΔE GFP (○). Titers expressed as plaque forming units/mL.

All YFV full length and replicon viruses had specific infectivity values within a comparable range (9.0×10^5^–1.8×10^6^/µg RNA) indicating that all molecular manipulations resulted in the generation of stable infectious YFV RNAs capable of initiating viral replication. All YFV variants were analyzed for their ability to grow and produce infectious particles following electroporation in BHK-21 or VEEV-YFV-17D-Help BHK-21 packaging cells respectively. YFVs 17D and Asibi containing a 5′ intra-ORF reporter expression cassette were replication competent following electroporation in BHK-21 cells. However, insertion of GFP or Cherry into YFV 17D ORF in a 5′ orientation resulted in a ∼24 h lag in replicative kinetics and an approximately ten fold decrease in peak titers (**Figure 4A**). Furthermore, RT-PCR analysis of Asibi GFP, using oligo-nucleotide primers which flanked the GFP expression cassette, indicated that although initially replication-competent in BHK-21 cells, this reporter cassette was relatively unstable, with a near complete loss of exogenous sequence being observed following a single passage in C6/36 cells (data not shown).

Interestingly this trend did not hold for the YFV 17D 5′ and 3′ envelope deletion replicons. Parallel electroporation of 17D 5′ ΔE Cherry and 17D 3′Δ GFP on BHK-21 and BHK-21 VEEV-YFV-17D-Help cells confirmed that the engineered sequence deletions resulted in the generation of suicide 17D genomes and that these genomes were only capable of spreading when full length E was provided in *trans*. For these replicons, similar growth kinetics and peak titers were observed for 17D 5′ ΔE regardless of the presence or absence of the Cherry coding cassette, while 17D 3′ ΔE displayed slightly lower peak titers than its respective GFP containing replicon (**Figure 4B**). Nevertheless, 17D deletion mutant replicons were observed to have similar growth and fluorescent protein expression kinetic profiles as compared to full length constructs, reaching peak titers by two to three days post-electroporation albeit to ≥100-fold lower peak titers.

### Optimization for detection and analysis of YFV 17D deletion mutant recombination

Previous analysis has repeatedly demonstrated that electroporation of BHK-21 cells with full-length flavivirus/alphavirus RNAs over a range of 10 µg to 10^−6^ µg results in ten-fold serial dilutions of electroporation efficiency (number of BHK-21 cells successfully transfected) when quantified using infectious centers assay (C.E. McGee unpublished). Electroporation of known concentrations of RNA into known numbers of BHK-21 cells could be correlated to an expected number of primary infections. Furthermore, analysis of viral recombination has suggested that the conditions for cell culture purification of putative recombinants are highly virus specific (C.E. McGee unpublished data). Experiments were performed to determine the optimal passage protocol and lower limit of detection of a full length YFV 17D against a saturated background (∼2×10^6^ cells 100% infected) of ΔE replicons. Briefly ten-fold serial dilutions of purified YFV 17D Cherry RNA (range: 10^1^–10^−6^ µg RNA) were co-electroporated in BHK-21 cells against 10 µg each 17D 5′ ΔE and 17D 3′ ΔE. One-fifth of the total volume of each electroporation (∼2.0×10^2^ cells in 2.0 mL total volume BHK-21 maintenance medium) was then plated in a single well of a six-well plate. At 24, 48, 72, and 96 h post-electroporation, total supernatant was harvested and replaced with sterile medium. Five hundred microliters were then used to inoculate C6/36 and Vero cell ∼80% confluent monolayers grown in six-well plates. Vero and C6/36 plates were examined daily for up to four dpi for signs of expanding Cherry foci indicating recovery of full length 17D Cherry. Analyses of detection sensitivity performed in this way indicated that Vero cells were ∼100-fold more sensitive than C6/36 cells for the detection of 17D Cherry when titrated against a saturated background of envelope deletion replicons (data not shown). Furthermore, it was determined that optimal detection was achieved at 72 h post-electroporation to a sensitivity of 10^−3^ µg 17D Cherry RNA input (equivalent to ∼50 infectious units as determined by infectious centers assay).

To determine if detection sensitivity could be further increased, 500 µL of electroporation supernatant samples from 17D Cherry dilutions 10^−3^–10^−5^, from the 72 h post-electroporation time point were used to infect 1.0×10^7^ Vero cells in suspension (as described above) followed by plating in 96-well plates. Analysis in this way increased the sensitivity of 17D Cherry detection to a dilution of 10^−4^ µg 17D Cherry RNA (∼5 infectious units) a level of sensitivity equivalent to that previously reported for a similarly designed “recombination trap” which successfully purified recombinant JEV [39]. Therefore, based on these analyses, it was decided that all attempts to recover 17D recombinant virus would involve co-electroporation of the recombinant cross (envelope deletion replicons) followed by a 72 h incubation at the appropriate growth temperature. Then recovery of recombinant viruses would be attempted by direct infection of 1.0×10^7^ Vero cells in suspension followed by plating in a 96-well format and incubation at 37°C with 5% CO_2_ for 72 h. Plates were then fixed, stained by IFA, and all wells visually examined for the presence of YFV 17D infectious foci which would be suggestive of the presence of a full length recombinant virus. 17D 5′ ΔE and 17D 3′ ΔE co-electroporation recombinant crosses (10 µg per RNA species) were performed in triplicate in both BHK-21 and C_7_10 cells. 17D 5′ ΔE Cherry and 17D 3′ ΔE GFP were also co-electroporated (10 µg per species) to allow for estimation of co-infection efficiency. Despite achieving estimated YFV replicon co-infection levels of ∼2.4×10^6^ in BHK-21 cells and ∼1.05×10^5^ in C_7_10 cells, no evidence of YFV 17D recombination was detected.

### Analysis of YFV superinfection resistance in Vero and C_7_10 cells

The most important requirement for the generation of a recombinant viral genome is spatial and temporal association of the donor and acceptor templates to allow for polymerase template switching. For that, a vertebrate or mosquito vector would have to either be simultaneously infected by two viruses, or superinfected with a second virus while harboring a primary infection, followed by sufficient replication of both genomes to allow for recombination, amplification, and subsequent selection to occur. Superinfection resistance studies were therefore performed to determine if primary infection with YFV Asibi would influence the replication of a secondary infection with YFV 17D. Primary infections were performed by electroporating RNA derived from a GFP expressing Asibi IC. (YFV-As RBZ GFP IC); this virus was chosen because it allowed for visual estimation of the percentage of cells positive for primary YFV infection, without requiring fixation. At seven days post-electroporation (≥95% of cells positive for GFP expression) cells were superinfected with virus derived from a 17D Cherry RBZ IC. This virus was chosen for these studies because the expression of the mCherry fluorescent protein would allow for easy distinction between 17D and Asibi during virus titration. Superinfection growth curves were also performed using Asibi GFP infected cells and an unrelated alphavirus, CHIK-LR-5′-Cherry. These infections were included to verify that any changes in 17D replication kinetics (if observed) were due to the establishment of YFV specific superinfection resistance and not a result of Asibi infection resulting in the induction of a non-specific antiviral state. Additionally, control (mock) electroporated Vero and C_7_10 cells were also infected with either 17D-Cherry or CHIK-LR-5′-Cherry in parallel.

**Figure 5:**
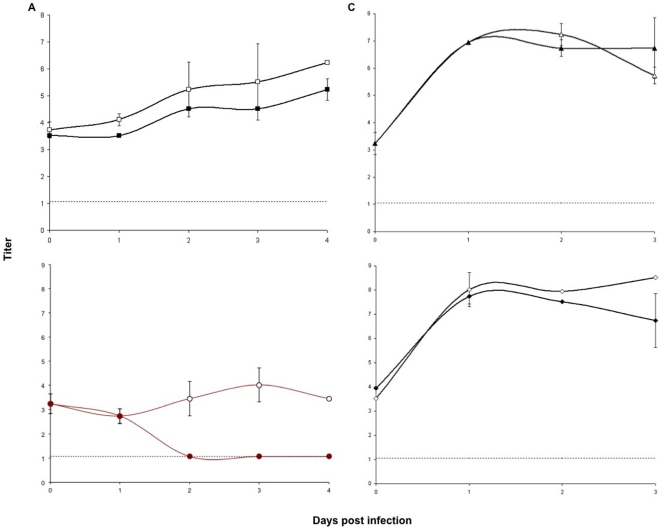
Superinfection of YFV Asibi GFP infected Vero and C_7_10 cells with either YFV 17D Cherry or CHIKV 5′ Cherry. A) YFV 17D-Cherry growth kinetics on naïve (□) and YFV Asibi GFP infected (▪) Vero monolayers. B) YFV 17D Cherry growth kinetics on naïve (○) and YFV Asibi GFP infected (•) C_7_10 monolayers. C) CHIKV 5′ Cherry growth kinetics on naïve (Δ) and YFV Asibi GFP infected (▴) Vero monolayers. D) CHIKV 5′ Cherry growth kinetics on naïve (◊) and YFV Asibi GFP infected (♦) C_7_10 monolayers. Titers expressed as log_10_TCID_50_/mL.

Average titers of 17D-Cherry harvested from Vero cells that were also infected with Asibi-GFP were approximately 0.5–1.0 log_10_TCID_50_/mL lower than from corresponding mock electroporated Vero cells at all times post-superinfection (**Figure 5A**). 17D Cherry was not detected (limit of detection 1.06 log_10_TCID_50_/mL) in Asibi GFP infected C_7_10 cells beyond 24 h post-infection, with titers at this time point likely representing residual viral inoculum. Average titers of 17D Cherry harvested from C_7_10 cells harboring a primary Asibi GFP were significantly (p<0.05) lower at two, three, and four dpi as compared to corresponding time points harvested from mock electroporated C_7_10 cells. Interestingly, primary infection with Asibi, in C_7_10 cells, appeared to result in the generation of a cellular environment that was completely refractory to superinfection with/replication of 17D (**Figure 5B**). As expected, previous infection with Asibi-GFP had no significant untoward effect on CHIK-LR-5′-Cherry replication in either cells line used (**Figure 5C,D**).

## Discussion

To date, all studies on RNA virus recombination can be categorized into two approaches: 1) comparative phylogenetic analysis of naturally occurring recombinant sequences, or 2) detection of recombinant progeny from experimental cell culture systems using high multiplicities of infection. In both cases, the recombinant genomes that have been observed have undergone extensive *in vivo/in vitro* replication and selection prior to sequence analysis. The nature of these RNA-dependent RNA polymerase template switches were then assessed retrospectively so that inferences could be made about the nature of the event which gave rise to the recombinant progeny. The vast majority of the evidence for mosquito-borne flavivirus recombination, generated in this way, has suggested that either genetic exchange occurs via homologous recombination or that these observed homologous recombination events were the result of the constraint on the need to reproduce an intact and unmodified ORF. Simply put, if the genetic filter for genome viability requires homologous-like recombination, that is all that will be detected in nature.

Homologous recombination of picornavirus oral polio vaccines is a well documented etiology of vaccine associated paralytic poliomyelitis [61]–[63]. It has been hypothesized that homologous flavivirus vaccine/vaccine or vaccine/wt recombination should be considered as a potentially significant impediment to the development of flavivirus live attenuated vaccines [34], [35], [44]. Given that these picornaviruses and flaviviruses have similar coding strategies involving a single stranded positive sense genome comprised of a single ORF, and, that naturally occurring recombination has been well documented for two genera of the family Flaviviridae (*Hepacivirus*, and *Pestivirus*), it may seem logical to assume that all viruses belonging to the genus *Flavivirus* might have similar propensities to undergo recombination. However, OPV intra-vaccine replication coincident with virus shedding has been observed to persist for ≥60 days post-vaccination [64]–[66], however post-immunization replication of YFV 17D does not persist for more than a few days and has been difficult to detect even during severe adverse events [67]. Furthermore, it has been noted that those strains of OPV that replicate longer in the host have a higher propensity for recombination [66]. Since it is logical to assume that recombination potential would be directly related to replication persistence, and OPV and YFV 17D display significant differences with regard to the duration of post-vaccination replication, direct extrapolation of the conditions that favor picornavirus recombination and its consequences with respect to flavivirus vaccine safety is an overly simplistic view.

However, arthropod-borne flaviviruses have long been known to cause persistent life long infections in arthropod vectors thus allowing for multiple transmission events to occur. Although vertebrate infection with these viruses is generally considered to be acute, several studies have demonstrated the potential for flaviviruses (genus *Flavivirus*) to establish persistent infection in vertebrates. This should perhaps not be that surprising considering the natural life-cycle of the hepaciviruses and pestiviruses involves persistent infections in humans and agricultural animals, respectively. Persistence has been documented for a number of flaviviruses including the un-vectored Modoc virus in hamster kidney cells [68],TBEV in non-human primates [69]–[74] and a number of Japanese encephalitis serocomplex viruses [75]–[79].

JEV and WNV have been shown to be capable of establishing persistent infection of the nervous system in humans and non-human primates with isolatable virus detected up to 5 ½ months after infection [76], [78]. Interestingly, WNV appears to have some specific propensity to establish persistent infection in the kidneys resulting in virus being shed in the urine [76], [77], [79]. In fact the detection of WNV RNA in human urine samples collected up to 6.7 years following primary infection suggests that persistent infection may result in the chronic symptomology experienced by some patients [79]. It should be noted that infectious virus has never been isolated from serum samples in any persistently infected animals/humans beyond the initial acute viremia (5–7 days post infection). Therefore the epidemiological implications in terms of the ability to facilitate recombination and natural arthropod-borne transmission remain unknown.

The studies described here were rationally designed to provide empirical data to assess the potential for flavivirus recombination using YFV 17D replicons bearing complementary in-frame E coding sequence deletions. The constructs were designed to strongly favor recombination, and the detection conditions were optimized to achieve high sensitivity recovery of putative recombinants. Electroporation of these constructs into BHK-21 cells both with and without a VEEV packaging construct, capable of supplementing functional envelope in *trans*, verified the viability and “suicide” phenotype of these replicons (data not shown). Interestingly, replicon particle titers harvested from packaging cells were ≥1000-fold less than as previously reported for YFV 17D replicon genomes [56]. Both of the deletion mutant replicons (17D 5′ ΔE and 17D 3′ ΔE) are competent for the expression of an E protein that has a large internal deletion. 17D 5′ ΔE contains an 80aa deletion within E dimerization domain (DII) including the fusion loop (aa 98–110), while the majority of the receptor binding domain (89aa of E DIII) has been removed from 17D 3′ ΔE. However, although major functional domains have been removed, these replicons have been designed in such a way that E truncated proteins should be expressed and maintain the correct transmembrane topologies of the native 17D E protein. It was possible that these truncated E proteins may have been incorporated into replicon containing virus-like-particles. If this were to happen, these particles would have significantly impaired infectivity due to their inability to interphase with cellular receptors and/or initiate fusion of the viral and endosomal membranes. Furthermore, a second possible explanation for these titer discrepancies may be that the presence of truncated E protein may in some way disrupt particle assembly.

McGee *et al.*, (2008) hypothesized that because flaviviral infection in a mosquito/tick is persistent, in nature recombination would most likely occur in the arthropod vector [80]. Indeed the persistent replication in a mosquito vector can be quite long-lived as *Ae. aegypti* mosquitoes experimentally infected with Asibi have been observed to fatally infect monkeys for up to 168 days post-infectious bloodmeal [81]. Since 17D and 17D chimeric vaccines exhibit an impaired ability to escape the mosquito midgut, recombination events occurring between a vaccine and wt flavivirus would have to occur with co-infected midgut epithelial cells. The mosquito midgut is estimated to be comprised of ∼10^4^ epithelial cells [82]. Analysis by Smith *et al.*, (2008) suggest that only a small sub-population of these cells, perhaps 100, are susceptible to primary infection with Venezuelan equine encephalitis virus, a conclusion which has been further supported by the observations of relatively low numbers of primary WNV midgut infections regardless of input titer [57], [83]. Taken together, these data suggest that, although virus may be capable of spreading cell-to-cell throughout the entire midgut in an infected individual, for any given mosquito-borne virus only a small sub-population of midgut epithelial cells may be susceptible to primary infection, although the actual population of susceptible cells may be different for each virus. Thus the levels of co-infection described here, specifically with regard to arthropod cells (electroporation of C_7_10 with 17D 5′ ΔE-Cherry and 17D 3′ ΔE-GFP RNAs reproducibly resulted in ≥10^5^ co-infections), are highly significant because they suggest that even if every cell within the mosquito gut were simultaneously co-infected with two distinct flaviviruses, a recombinant virus would still not likely be produced.

Seligman and Gould (2008) recently argued that the predominant risk for flavivirus recombination was in the vertebrate host, given the potential for wt persistence and adverse vaccine replication. However, *in vivo* YFV homologous interference (superinfection resistance) was noted by Hoskins (1935) who observed that simultaneous or subsequent (within 48 h) inoculation of *Macacus rhesus* with YFV viscerotropic (Asibi) and neurotropic strains could ameliorate the lethal phenotype of YFV Asibi [84]. Since no detailed molecular techniques were available at that time it is difficult to determine if this resulted from superinfection resistance at the cellular level or via some immune/interferon mediated mechanism. Nevertheless, these data argue that if a natural co-infection were to happen during the course of vaccination uncontrolled dual replication would not likely occur. Given that vertebrate hosts can facilitate high titer replication of wt flaviviruses the potential for acute high level co-infection to facilitate YFV 17D recombination in vertebrate cells was examined. Co-electroporation of 17D 5′ ΔE-Cherry and 17D 3′ ΔE-GFP RNAs was observed to result in high level co-infection in BHK-21 (≥10^6^) cells as indicated by cells positive for the expression of both GFP and Cherry. Despite this artificially high level of co-infection at the cellular level and a lower limit of detection of ∼5 infectious units, no recombinant full length YFV 17D was detected. As such, these data strongly argue that even if acute robust post-vaccination replication were to occur within a vaccinee, recombination is unlikely. Furthermore given that recombination is a replication driven mechanism resulting from a template switching phenomenon of the viral polymerase and all ChimeriVax™ vaccine genomes derived their replicative machinery from the parental YFV 17D genome it is reasonable to expect similar recombination efficiency, or lack thereof, for these viruses.

A similarly designed study, described by Taucher *et al.*, (2009), also failed to detect recombinant tick-borne encephalitis virus (TBEV) and West Nile virus (WNV). Furthermore, recent analyses of YFV 17D *trans*-complementing pseudoinfectious genomes also failed to detect the generation of any viable recombinant full length virus despite sustained serial passage [56], [85]. It should be noted, however, that specific sequence manipulations were engineered into the YFV 17D genomes used by Shustov *et al.*, (2007; 2010) to decrease sequence homology specifically with the goal of prevention of recombination. Although the study by Taucher *et al.*, (2009), did not result in TBEV or WNV recombination, it did provide the first experimental evidence of the generation of viable flavivirus recombinants under extreme laboratory conditions using a Japanese encephalitis virus (JEV) replicon/replicon “recombination trap” [39]. In these studies, persistent high level co-infection, via serial passage in BHK cells, of *trans*-complementing JEV genomes was required to yield full length virus [39]. JEV recombinants isolated from this system were aberrantly homologous and contained sequence duplications. Cross-overs were observed to occur within the NS1 gene resulting in duplications of NS1 and both structural cassettes including their in-frame structural deletions. Analysis of the attenuated *in vitro* growth characteristics of these recombinants as compared to wt suggested that under a natural co-infection these recombinants would not be fit for selection relative to the parental viruses.

The lack of recombination of YFV using 17D in our studies as compared to JEV may be due to differences between the topologies of our replicons and those used by Taucher and coworkers. The 17D replicons used for our studies contained specific internal deletions of E coding sequences, while JEV replicons described by Taucher *et al.*, (2009) contained major deletions of different proteins, C and E. Thus, while our system required recombination to occur within the coding sequence of a single protein, the JEV system employed by Taucher *et al.*, (2009) allowed for expression of both truncated and full length C and E from a single covalently linked genome. Therefore, it is possible that the efficiency of generating a viable recombinant within the 17D system was less than that using the JEV system. A second possible explanation is that particular species of mosquito-borne flaviviruses may simply have different propensities to participate in recombination, indeed WNV and TBEV systems designed using similar topologies to the JEV recombinant cross did not generate viable recombinant virus. Furthermore, it is possible that the ability for different viruses to undergo recombination and/or for recombinants to be detected may be highly influenced by the specific cell culture conditions employed. Interestingly, even given its apparent propensity to recombine, the generation of JEV recombinants required three to five passages in cell culture. These data suggest that the efficiency of flavivirus recombination may be extremely low and in fact may require long-term sustained or persistent co-infection to allow for sporadic template switching to occur. Although such infections are known to occur for pestiviruses, via an immunotolerance mechanism, they appear to be unlikely for flaviviruses because of the acute nature of flavivirus infection in vertebrate hosts. Given the mounting experimental evidence against homologous recombination of flaviviruses both vaccine (17D) and wt (WNV, JEV, TBEV) as reported here and by Taucher and coworkers it is logical to conclude that in general arthropod-borne flaviviruses do not homologously recombine under acute co-infection circumstances and those extremely rare events that generate aberrant genomes are rapidly selected out of the viral population due to their decreased relative fitness.

Distinct similarities in post-recombination genome topologies can be observed for CHIKV intra-genic recombinants reported here and those of JEV recombinants as reported by Taucher *et al.*, (2009) (i.e. both contained non-homologous duplications of truncated structural genes). This supports our ability to extrapolate between alphavirus and flavivirus systems to draw general conclusions regarding RNA arbovirus recombination. Flavivirus recombination, like alphavirus recombination, when examined in the laboratory, appears to occur by non- homologous or aberrantly homologous template switching. This is perhaps not surprising giving that the likelihood of polymerase disassociation and re-initiation at specific homologous sites would require a high level of precision and perhaps facilitation by accessory proteins, none of which have been identified. However, reports of naturally occurring mosquito-borne flavivirus and alphavirus recombinants suggest that these viruses may undergo precisely homologous recombination in nature, with no aberrant sequence duplications, insertions, or deletions.

A critical analysis of the phylogenetic reports of DENV recombination suggests that not only is recombination occurring in nature, but that multiple completely homologous polymerase jumps are possible, and can result in the generation of mosaic DENV genomes [31], [35]–[37]. In fact, one report by Chen *et al.*, (2008) has identified a DENV 1 genome with three recombinant regions coincident with six homologous polymerase template switches.

We propose that the assertion that a flavivirus can undergo multiple homologous template switches and produce a recombinant progeny that is viable during a single co-infection represents a completely naïve view with respect to the nature of the molecular interactions involved. Working with CHIKV which seems highly amenable to recombination, or to a significantly disrupted gene order that allows for a much lower frequency recombination event to produce viable progeny, we have quantified the rate of intra-genic recombination to require at least 10^5^ co-infected cells to facilitate a single template switching event. If we were to extrapolate the conditions required to achieve six polymerase jumps, even if the nature of those switches (homologous vs. non-homologous) is disregarded, then ∼10^30^ co-infections would be required. Given that the suggested incidence of an adverse flavivirus vaccine event while simultaneously experiencing a wt infection has been estimated at 1 in 300 million [86] then the possibility of even a double homologous template switch during acute replication is highly unlikely.

Although, the molecular interactions that facilitate flavivirus recombination at the molecular level appear to be extremely inefficient there must be some finite possibility for such an event to occur should two functional templates exist in a single cell. In the recombinant crosses reported here this is achieved via electrical transfection of both genomes. However, in nature a single cell must be infected via virion-receptor mediated events with two viruses either simultaneously or more likely sequentially. As such, this represents a second potential obstacle to recombination. Intra-mosquito reports of flavivirus superinfection resistance are scarce. One report by Sabin (1952) suggests that *Ae. aegypti* mosquitoes harboring a primary infection with DENV become resistant to superinfection with YFV [87]. However, because no data regarding the number of individual mosquitoes assayed were provided the extent of this resistance is unclear. *In vitro* analyses of DENV co- and superinfection indicate that the suppression of heterotypic replication can be induced very rapidly, within 20 h post-primary infection [88], [89]. This type of resistance can be relatively long lived as DENV persistently infected C6/36 cells have been observed to produce significantly lower titers of a secondary challenge virus even when infections were separated by 50 days [90]. A recent study of WNV superinfection has begun to provide some insights into the mechanism of flavivirus superinfection resistance [91]. Zou *et al.*, (2009) demonstrated that replication of WNV was significantly decreased in cells harboring a persistently WNV replicon infection. Interestingly, this resistance could be overcome by specific mutations in E and NS4A which appeared to increase particle-receptor binding interactions and the affinity for interactions with host factors required for replication. Therefore, it was suggested that flavivirus superinfection resistance may result from sequestering of critical host factors by the replicative complex of the primary virus, which exists in abundance relative to the secondary virus [91].

To determine if a similar phenomenon could be observed for YFV, Vero and C_7_10 cells were superinfected with YFV 17D following primary infection with YFV Asibi. While Asibi infected C_7_10 cells appear to become completely refractory to 17D infection/replication superinfected Vero cells appear to be fairly permissive for 17D replication. It was noted by Zou *et al.*, (2009) that WNV superinfection resistance could be overcome by selection for viruses containing mutations which increased receptor binding affinity and RNA replication kinetics [91]. Indeed YFV 17D is a highly adapted to vertebrate cell culture [92] and as such may possess some, as of yet, unidentified mutations which allow for escape from superinfection resistance in vertebrate cells. The ability of CHIKV to replicate to similar titers and at similar kinetics in both Vero and C_7_10 cells harboring a primary infection with YFV Asibi suggest that primary YFV infection does not induce a non-specific antiviral state in these cell types.

Taken together the observations as reported here and those published by others[39] have repeatedly demonstrated that: 1) the safety and efficacy of live attenuated and chimeric live attunated flavivirus vaccines [43], [93] 1) if a recombinant flavivirus does arise following acute dual infection in a vertebrate host then it would likely be attenuated and likely be selected against, 2) if an arthropod vector was to become exposed to two distinct flaviviruses, the likelihood of sequential co-infection and/or recombination is extremely remote, and 3) even if what would be considered “worst-case” post-recombination genome topology were to occur, vaccine safety would not be compromised [80], [94].
